# Modular Organization of Muscle Synergies to Achieve Movement Behaviors

**DOI:** 10.1155/2019/8130297

**Published:** 2019-11-15

**Authors:** Kunkun Zhao, Zhisheng Zhang, Haiying Wen, Zihan Wang, Jiankang Wu

**Affiliations:** ^1^School of Mechanical Engineering, Southeast University, Nanjing, Jiangsu 211189, China; ^2^CAS (Nanjing) Digital Health Industrial Technology Research Institute, Nanjing, Jiangsu 210046, China; ^3^School of Electronic, Electrical Communication Engineering, University of Chinese Academy of Sciences, Beijing 101407, China

## Abstract

Muscle synergy has been applied to comprehend how the central nervous system (CNS) controls movements for decades. However, it is not clear about the motion control mechanism and the relationship between motions and muscle synergies. In this paper, we designed two experiments to corroborate the hypothesis: (1) motions can be decomposed to motion primitives, which are driven by muscle synergy primitives and (2) variations of motion primitives in direction and scale are modulated by activation coefficients rather than muscle synergy primitives. Surface electromyographic (EMG) signals were recorded from nine muscles of the upper limb. Nonnegative matrix factorization (NMF) was applied to extract muscle synergy vectors and corresponding activation coefficients. We found that synergy structures of different movement patterns were similar (*α*=0.05). The motion modulation indexes (MMI) among movement patterns in reaching movements showed apparent differences. Merging coefficients and reconstructed similarity of synergies between simple motions and complex motions were significant. This study revealed the motion control mechanism of the CNS and provided a rehabilitation and evaluation method for patients with motor dysfunction in exercise and neuroscience.

## 1. Introduction

A large amount of research has reported that the CNS uses a dimensionality reduction pattern to coactivate a set of motion primitives (MP) to achieve daily activity living (DAL). However, motor control is redundancy and we could achieve a specific motion by combining various activation muscles [1]. How the CNS selects primitives from a vast pool and achieves movement behaviors is a complicated issue in the field of movement neuroscience and neurorehabilitation.

Modularity or muscle synergy as a building block, both structural and computational, exhibited feasibility for achieving motion control [2]. It has been proven in animal experiments, such as in cats, frogs, and monkeys. d'Avella [3] analyzed the movements of frogs during jumping, swimming, and walking in naturalistic conditions and found three shared and two task-specific muscle synergies across behaviors. Research in rhesus macaques showed that the grasping and transporting movements were achieved by modulating the muscle synergies [4, 5]. Ting and Macpherson [6] analyzed the postural and balance control of cats and found that muscle synergies were correlated to the movement direction and endpoint force.

The modularity of motion control was also found in human motions. The study in various human locomotions, consisting of walking and running at different speeds, walking forward or backward, and walking under different loading conditions and different styles (rectilinear and curvilinear trajectories) [7, 8], showed that motions were driven by combining a few muscle synergy primitives. Shared and task-specific muscle synergies were also found in human locomotion [9, 10]. And, Barroso found muscle synergies merging in human walking and cycling [11], and cycling synergies are a linear combination of walking synergies. In the clinical research of stroke, decomposing and merging were more evident [12]. Comparing to the lower limb, the upper limb movement is more complicated and exquisite. d'Avella et al. [13, 14] analyzed and summarized the movements of point-to-point at different speeds, loads, forearm postures, and via-point movements, showing that the CNS achieved a goal by combining a set of building blocks (muscle synergy primitives). Israely et al. [15] studied the muscle synergy modulation function of hand-reaching tasks from different directions and found a representative set of synergies, which were from the muscle synergies extracted from the center of the reaching space, which could be modulated to achieve motions in different directions. Similar results were found in poststroke [12, 15]. However, there was little research on the relationship between movement patterns and muscle synergies. Besides, the relationship between simple motions and complex motions is ambiguous. A deeper understanding of motion control in modularity is necessary.

In this paper, we would mainly analyze the relationship between muscle synergies and motion primitives. Based on the prior research, two hypotheses are tested: (1) any motion can be decomposed to motion primitives, which are driven by muscle synergy primitives and (2) variations of motion primitives in direction and scale are modulated by activation coefficients rather than muscle synergy primitives.

## 2. Materials and Methods

### 2.1. Subjects

Twenty-eight subjects with no neurological injury (male: eleven, female: seventeen, and age: 23.68 ± 1.74 years) were recruited for the study. All subjects are right-hand dominant. They were informed about the procedure and possible discomfort before giving their informed consent. The research was approved by the ethical committee of the university.

### 2.2. Experiment Procedures

Two experiments were designed for corroborating hypotheses. The first experiment (E1) consisted of three simple upper limb motions (SM) and five complex upper limb motions (CM). The simple motions included shoulder flexion/extension, shoulder abduction, and elbow flexion/extension. The complex motions covered touching head in the sagittal plane and the frontal plane, respectively, putting one hand behind the back, and shoulder pushing up in the sagittal plane and the frontal plane, respectively. All participants stood in the anatomic pose. More detailed motion information was illustrated by Pan et al. [12]. All subjects participated in the E1. The second experiment (E2) [16] was carried out in eleven male subjects. The subjects for E2 were instructed to execute reaching movements in six directions and three distances in a horizontal plane in a seating pose. Repeating the procedure six times for every reaching movement, 108 (3 × 6 × 6) trials were performed for every subject.

### 2.3. Data Collecting and Preprocessing

Surface electromyographic (EMG) signals were recorded (Trigno Wireless EMG System, Delsys, USA) from nine dominant muscles of the right upper limb, including triceps brachii long and lateral head (TriLong and TriLat); pectoralis major (Pecm); deltoid anterior, medial, and posterior (DeltA, DeltM, and DeltP); trapezius upper (TrapUpper); biceps brachii (Bic); and brachioradialis (Brad). Electrodes were placed longitudinally along with the muscle fiber direction on corresponding muscles based on the guidelines of the Surface Electromyography for the Noninvasive Assessment of Muscles (SENIAM) [17].

Before the EMG processing, we eliminated the recordings contaminated due to disturbance and noise. Then, raw EMG signals were high-pass filtered (5^th^ order Butterworth filter, the cutoff frequency of 50 Hz), zero-meaned, rectified, low-pass filtered (5^th^ order Butterworth filter, the cutoff frequency of 5 Hz), and integrated over 20 ms [3, 11, 16]. To facilitate comparison across subjects, the EMG envelope was normalized by the average of the top 10 maximum of each muscle from every individual [11].

### 2.4. Data Analysis

#### 2.4.1. Extracting Muscle Synergies

Muscle synergy theory assumes that EMG patterns can be described as a linear combination of a set of muscle synergies (time invariant) activated by corresponding activation coefficients (time variant). It can be described as follows:

(1) $$\begin{array}{c} E_{m\times t}=W_{m\times n}C_{n\times t}+e_{m\times t}, \end{array}$$

where *E*_*m*×*t*_ is the preprocessed EMG, *m* is the number of muscles, and *t* is the number of sampling. *W*_*m*×*n*_ specifies the spatial profiles of activation, named the muscle synergy matrix, *n* is the number of muscle synergies. *C*_*n*×*t*_ is the activation coefficient, which is time varying. *e*_*m*×*t*_ is the error of reconstruction. We applied the nonnegative matrix factorization (NMF) [18] to extract muscle synergies. To avoid *W* and *C* converge to a local minimum, we repeated 50 times for each synergy.

#### 2.4.2. Determining the Minimum Number of Muscle Synergy

The NMF algorithm starts with an initialized *n*. We increased the number of muscle synergy from one to nine. Reconstruction quality was evaluated by calculating the variance accounted for (VAF) [14]. The structure of muscle synergy was affected by the minimum number of synergy. To reconstruct the EMG patterns better and decrease the dimensionality of the muscle synergies, two criteria were applied. Criteria 1: the minimum of muscle synergy was defined as the number that the total VAF was greater than 95% [12] (in the E2, VAF > 90% [19]) and criteria 2: an additional synergy did not contribute more than 5% in the reconstruction of the EMG envelope. The VAF is defined as follows [20]:

(2) $$\begin{array}{c} \text{VAF}=1-\frac{\sum_{i=1}^{m}\sum_{j=1}^{t}{\left(e_{i,j}\right)}^{2}}{\sum_{i=1}^{m}\sum_{j=1}^{t}{\left(E_{i,j}\right)}^{2}}. \end{array}$$

#### 2.4.3. Evaluating the Similarity and Merging the Synergies

Before calculating the synergies similarity, we first matched the muscle synergy vectors from all synergy sets adopting the Hungarian algorithm [21]. The Pearson correlation coefficient (*r*_*i*,*j*_) and cosine similarity (*s*_*i*,*j*_) were used to assess the similarity of synergy vectors. To identify how the synergies extracted from complex motions were reconstructed by the linear combination of synergies extracted from simple motions, we applied the algorithm proposed by Cheung et al. [22], in

(3) $$\begin{array}{c} w_{i}^{\text{CM}}=\overset{n_{\text{SM}}}{\underset{k=1}{\sum }}m_{k}^{i}w_{k}^{\text{SM}},\;\;m_{k}^{i}\geq 0,\;i=1,\ldots ,n_{\text{CM}}, \end{array}$$

where *w*_*i*_^CM^ is the *i*th synergy vector from a complex motion, *w*_*k*_^SM^ is the *k*th synergy vector from simple motion, and *m*_*k*_^*i*^ is a nonnegative coefficient which denotes contributions of the *k*th synergy vector from simple motion for the structure of *i*th synergy vector from the complex motion.

#### 2.4.4. Motion Modulation Indexes (MMI)

In the E2, we executed reaching movements in different directions and distances. To assess the modulating extent of activation coefficients among motion patterns, the motion modulation indexes were applied. We applied two indexes, root mean square of modulating signals (RMS-MS) and the VAF of synergy (VAF-Syn). RMS-MS represented an absolute activation degree, and VAF-Synergy (VAF_*i*_^syn^) showed a relative activation degree. The two indexes could give an objective description of modulation. For one synergy, the VAF_*i*_^syn^ is defined as [16]

(4) $$\begin{array}{c} {\text{VAF}}_{S_{i}}=\frac{\sum_{i=1}^{m}\sum_{j=1}^{t}{\left(WC-w_{i}c_{i}^{T}\right)}^{2}}{\sum_{i=1}^{m}\sum_{j=1}^{t}{\left(E_{i,j}\right)}^{2}},\\ {\text{VAF}}_{i}^{\text{Syn}}=\frac{{\text{VAF}}_{S_{i}}}{\sum_{i=1}^{N}{\text{VAF}}_{S_{i}}}, \end{array}$$

where *w*_*i*_ is the *i*th muscle synergy and *c*_*i*_^*T*^ is the corresponding activation coefficient. VAF_*S*_*i*__ and VAF_*i*_^syn^ are the VAF and the synergy VAF of the *i*th muscle synergy, respectively.

## 3. Results

### 3.1. Analysis of E2

#### 3.1.1. Extracting Muscle Synergies

**Figure 1:**
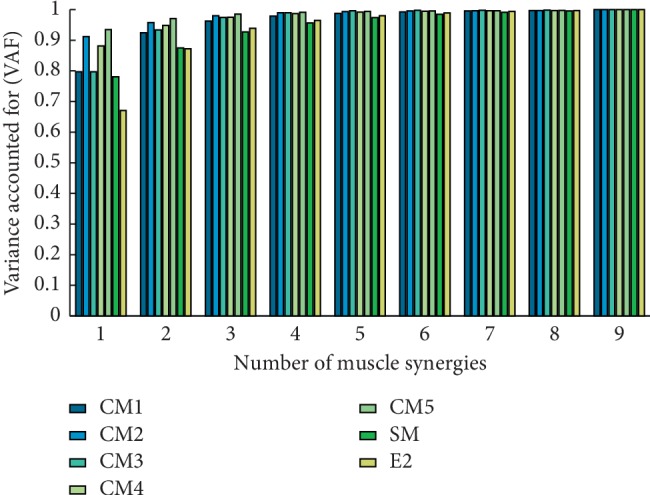
The variance accounted for (VAF) with respect to the number of muscle synergies. Colored bars indicate different motion patterns, respectively. Muscle synergies were extracted by the NMF algorithm. For reaching movement (E2), three synergies were extracted from the concatenated ENG. We extracted four synergies from concatenated simple motions (SM). 3, 2, 3, 2, and 2 synergies were extracted from five complex motions (CM1, CM2, CM3, CM4, and CM5), respectively.

**Figure 2:**
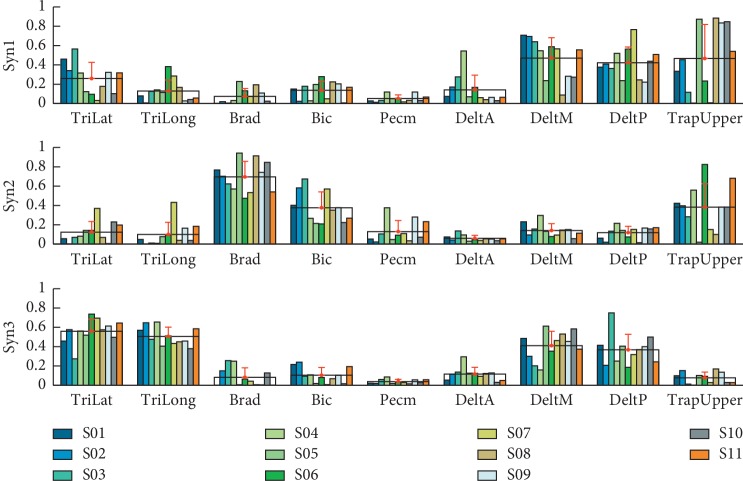
The structure of muscle synergies extracted from reaching movement. Colored bars indicate different subjects (11), and each muscle is shown in a group. Black wireframes and red bars are the group mean and standard deviation, respectively.

The VAF is shown in Figure 1. According to the criteria described above, we identified three synergies (2.82 ± 0.40, corresponding VAF is 0.94 ± 0.01) in reaching movement (E2) for further analysis.  Figure 2 exhibits three synergy structures extracted from preprocessed and pooled EMG data. Every synergy activated certain muscles corresponding to the upper limb motion. The first synergy mainly drove the shoulder flexion/abduction and internal rotation (Pecm and DeltA) and elbow flexion (Bic). The second synergy typically involved the movement of elbow extension (TriLat and TriLong), shoulder abduction (DeltM and DeltP), shoulder external rotation (DeltP), and shoulder extension (TriLat, TriLong, and DeltP). The third synergy covered the elbow flexors (Brad and Bic) and TrapUpper.

The cosine similarity (*s*) and the Pearson correlation coefficient (*r*) between synergy vectors from all subjects are shown in Table 1. Results showed that the parallel synergy vector in all subjects was relevant. *t*-test results also illustrated that there was no significant difference among synergy vectors (*α*=0.05).

#### 3.1.2. Similarity Analysis in Different Directions and Distances

**Figure 3:**
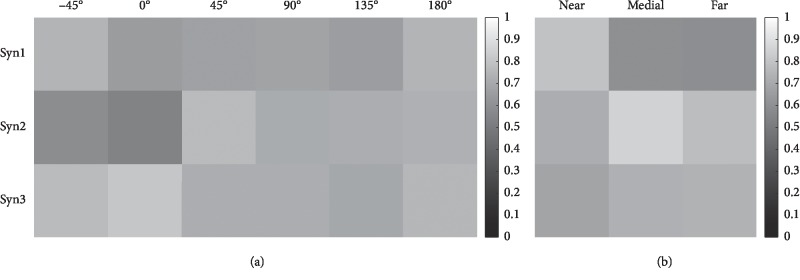
*t*-test results from six directions (a) and three distances (b) among all subjects. The samples are 55 (*C*_11_^2^). The deeper color indicates a smaller *p* value.

To compare the variance of synergy vectors from different directions and distances, *t*-test was performed.  Figure 3 shows the mean *p* values of the *t*-test among directions (a) and distances (b). Results showed that all *p* values were greater than 0.54 in directions (Table 2) and 0.59 in distances (Table 3), indicating that the synergy vectors from all directions and distances were from a population. Then, we concatenated the data from all directions and distances, respectively. The *t*-test results showed that there was also no significant correlation in all directions and distances. For every synergy in six directions (Table 2), the average *p* values are 0.71 ± 0.21, 0.70 ± 0.21, and 0.73 ± 0.19 (*α*=0.05, *n* = *C*_66_^2^ = 2145). And for every synergy in three distances (Table 3), the corresponding average *p* values are 0.69 ± 0.24, 0.78 ± 0.16, and 0.72 ± 0.19 (*α*=0.05, *n* = *C*_33_^2^ = 528), respectively.

#### 3.1.3. Motion Modulation Indexes (MMI)

**Figure 4:**
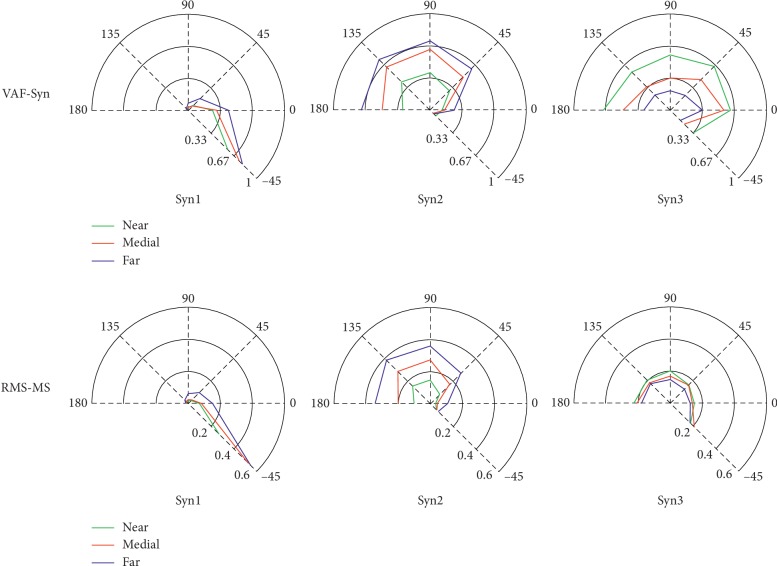
The radar map of MMI for three synergies (Syn1, Syn2, and Syn3). The first row is the VAF of synergy (VAF-Syn), and the second row is the root mean square of modulation signals (RMS-MS). Each column corresponds to a synergy. The radar map shows the six directions (−45, 0, 45, 90, 135, and 180), and colored lines indicate different distances (near, medial, and far).

The MMI for every direction and distance are shown in Figure 4. We found that every synergy was activated mainly in certain directions or distances. For example, the first synergy was activated mainly in 0 and −45 direction. The second synergy was in the direction of 45, 90, 135, and 180. The third synergy was activated in all directions. In terms of distances, the first two synergies showed similar trends, i.e., the farther the distance, the greater the MMI. However, the third synergy exhibited the inverse characters, i.e., the farther the distance, the smaller the MMI. Two criteria, VAF-Syn and RMS-MS, demonstrated similar characters.

### 3.2. Analysis of E1

#### 3.2.1. Extracting Muscle Synergies

**Figure 5:**
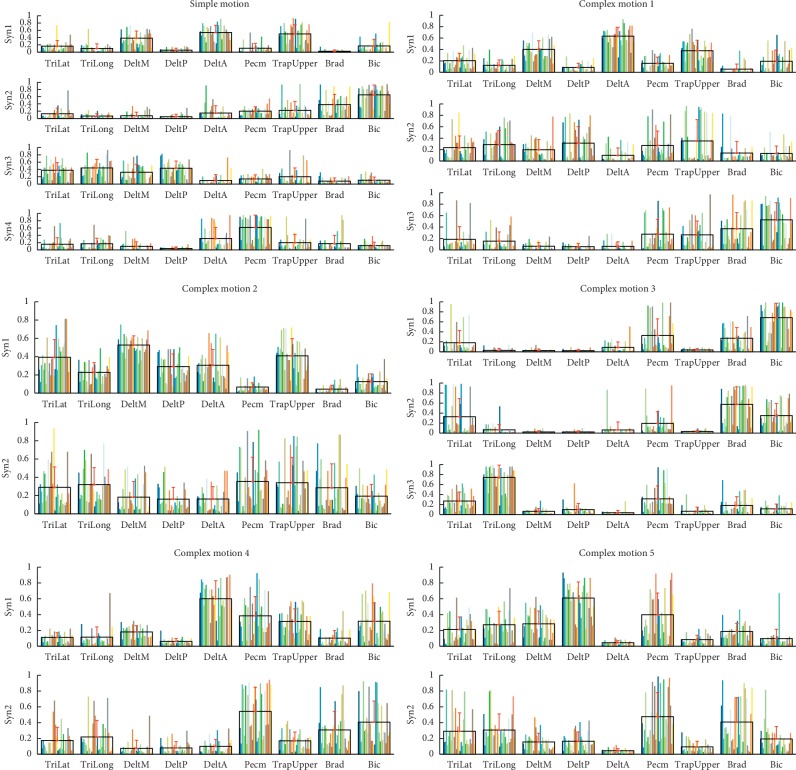
Synergy structures extracted from concatenated simple motion and complex motions. Four synergies were extracted from simple motion, and 3, 2, 3, 2, and 2 synergies were extracted from five complex motions, respectively. Colored bars indicate different subjects (28 subjects for E2). Black wireframes are group means, and red bars are standard deviation.

For E1, we preprocessed the raw EMG data according to the abovementioned methods. However, the threshold was set 0.95 for determining the number of minimum synergies. Four synergies were selected for simple motion and 3, 2, 3, 2, and 2 synergies were identified for every complex motion, respectively (Figure 1): more specifically, 4.11 ± 0.63, 3.11 ± 0.50, 2.21 ± 0.57, 2.71 ± 0.66, 2.43 ± 0.57, and 1.82 ± 0.55. The spatial structure of muscle synergies from every motion pattern is shown in Figure 5. For simple motion, the first synergy mainly activated shoulder abductor (DeltA and DeltM) and TrapUpper, which drove the motion of shoulder abduction. The second synergy primarily stimulated elbow flexor (Bic and Brad), which actuated the motion of elbow flexion. The third synergy drove the shoulder and elbow extension (TriLat, TriLong, DeltM, and DeltP). The forth synergy led to the motion of shoulder flexion (Pecm and DeltA). For every complex motion, every synergy chiefly activated certain muscles also, which actuated similar upper motions.

**Figure 6:**
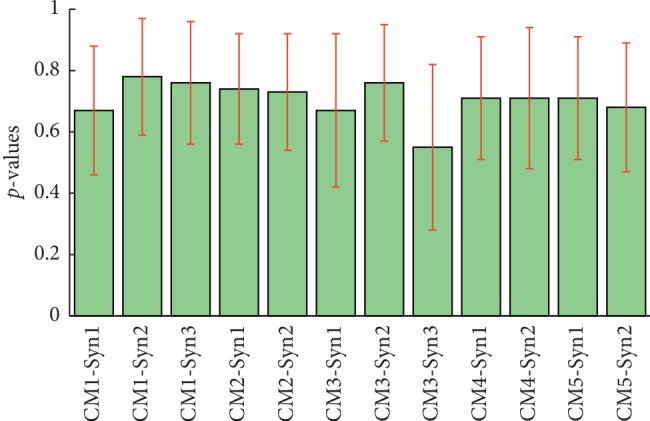
The synergy similarity among subjects from complex motions. The red bar indicates the standard deviation. CM1-Syn1 means the first synergy from complex motion 1. Other horizontal axis labels are similar.

*t*-test analysis was conducted among the subjects (*α*=0.05). The synergy similarity among subjects is shown in Figure 6. The results rejected the null hypothesis (the sample data come from a population). We analyzed the synergy similarity of all complex motions given in Table 4. A paired synergy is considered significantly correlated if the *p* is >0.5. We found that the synergy structure exhibited correlations between CM1 and CM2, and CM4 and CM5. However, the first synergy from CM4 and CM5 displayed a negative correlation.

#### 3.2.2. Analysis of Synergy Similarity between Simple Motion and Complex Motions

**Figure 7:**
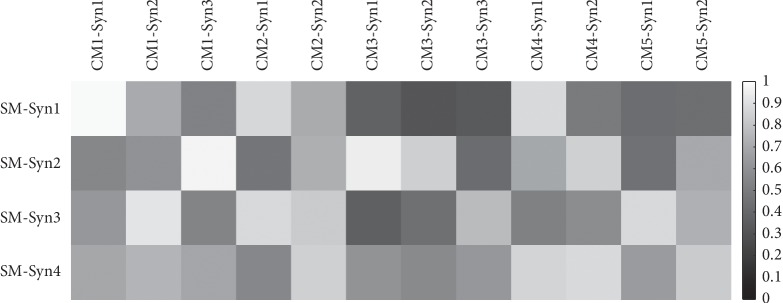
Cosine similarity of synergies between simple motion and complex motions. CM1-Syn1 means the first synergy from complex motion 1. SM-Syn1 expresses the first synergy from simple motion. Other abbreviations are semblable. A deeper color means a lower similarity.

The similarity of every extracted synergy between complex motions and the simple motion was computed by cosine similarity (Figure 7).  Table 5 shows the average of synergy similarity that every complex motion relates to simple motion. The higher similarity was found between the synergies from the complex motions and synergies from simple motion which composed the complex motion. For instance, touching head in the sagittal plane (CM1) consists of shoulder and elbow flexion. And, we found a greater similarity in the CM1-Syn1 and SM-Syn1, and CM1-Syn3 and SM-Syn2, which was similar in the synergy structure analysis (Figure 5: complex motion 1). We also found the merging process between simple motion and complex motions.  Table 6 demonstrates the merging coefficients (Mer-Coe) and reconstructed similarity (ReSim). We considered that the merging process was significant when the Mer-Coe was higher than 0.3 [11]. The results displayed that all reconstructing similarity was higher than 0.8.

## 4. Discussion

The purpose of this study was to investigate the relationship between muscle synergies and motion primitives of the upper limb motions. In previous studies, two muscle synergy models, time-varying synergy and time-invariant synergy, were used to analyze muscle patterns [13, 23]. In our study, we extracted time-invariant muscle synergies adopting the NMF algorithm. Two experiments were designed, reaching movements and simple/complex motions. According to the criteria predefined, we found three synergies were sufficient to explain >90% of the total variability of EMG activity of the nine studied muscles for reaching movements from eleven subjects. In the study of reaching movements of different conditions, three to five time-varying muscle synergies were extracted [24, 25]. The results were coincident with the prior study. For simple motion and complex motions, we just selected one or two synergies in the threshold of 0.9, which could not describe the spatiotemporal structure of the synergy. Thus, the threshold was set to 0.95, and we selected 4, 3, 2, 3, 2, and 2 synergies, respectively.

The study discussed the influence of directions and distances to muscle synergy in reaching movements (E2). *t*-test analysis (Tables 2 and 3) showed that synergies in different directions and distances were irrelevant. In the analysis of MMI, we found that RMS-MS and VAF-Syn exhibited resembled distributions in the radar map (Figure 4). The main performance was that the first synergy was activated mainly in the right and right rear motions. The second synergy mainly involved front, front-left, and left motions. However, the third synergy covered all directions. In the analysis of distance, the first two synergies displayed similar characters, farther distance with greater MMI. However, the third synergy showed an inverse character. We speculated that the third synergy was a shared synergy structure for reaching movements. The conjecture was verified by analyzing the synergy structure (Figure 2). The results revealed that the CNS controlled the motions in different patterns (directions and distances) by adaptively modulating the corresponding activation coefficients.

The E1 mainly analyzed the muscle synergy patterns of the simple motion and complex motions. *t*-test analysis showed synergy coincidence among subjects. However, there was one case which rejected the null hypothesis in CM3-Syn1, CM3-Syn2, CM3-Syn3, and CM5-Syn1, respectively (*n* = *C*_28_^2^ = 378). Considering the interference and noise of EMG signals, we thought the results were reliable.

The CM1 (touching head in the sagittal plane) could be decomposed into shoulder and elbow flexion. And, the CM2 (touching head in the frontal plane) included the simple motions of shoulder abduction and elbow flexion. The analysis of synergy similarity between CM1 and CM2 showed a positive correlation (>0.5) in the corresponding synergy. The synergy structure of the two motions (Figure 5) also displayed analogy. Semblable results were observed between CM4 and CM5. The results coincided with the conclusion in E2.

The similarity of synergy vectors was analyzed between simple motion and complex motions. The results verified that the CNS controlled the motions by recruiting a set of muscle synergy primitives. Combining the study of reaching movements in E2, we knew that every muscle synergy pattern corresponded to a motion primitive.

As a quantitative assessment tool, muscle synergy has been used widely in motor neuroscience and rehabilitation neuroscience. However, the raw EMG signals are contaminated easily, and various preprocessing methods increase the difficulty to compare among researchers. Researchers have reported that experiment conditions have an effect on the envelope of the EMG, including speed, load, and posture [13]. In further work, we could study how the synergy modulates the motion in more conditions.

## 5. Conclusions

This study presented the possible patterns of the CNS controlling motions by two experiments, reaching movements in a horizontal plane and simple/complex motions. We applied the NMF to extract muscle synergies. Similarity analysis and *t*-test in muscle synergies indicated that the CNS modulated activation coefficients to achieve different motion patterns. Besides, for a complex motion, which included several motion primitives, the CNS recruited a set of muscle synergy primitives which drove the corresponding motion to coactivate the motion. Our results provided an interpretable strategy for the CNS controlling the motions. This would be a potential implication for evaluating and making rehabilitation plans in rehabilitation neuroscience.

## Figures and Tables

**Table 1:** Cosine similarity (*s*) and Pearson correlation coefficient (*r*) among synergy vectors from all subjects and the *t*-test results. The null hypothesis is that the synergy vectors come from a population (*α*=0.05, *N* = *C*_11_^2^ = 55).

Synergies	*s*	*r*	*t*-test
Syn1	0.94 ± 0.04	0.91 ± 0.07	0.56 ± 0.24
Syn1	0.93 ± 0.04	0.81 ± 0.12	0.80 ± 0.16
Syn1	0.85 ± 0.09	0.73 ± 0.16	0.73 ± 0.19

**Table 2:** *t*-test results from six directions (*n* = 2145, *α*=0.05).

Synergies	−45°	0°	45°	90°	135°	180°
Syn1	0.75 ± 0.18	0.65 ± 0.21	0.67 ± 0.19	0.68 ± 0.28	0.66 ± 0.27	0.75 ± 0.24
Syn2	0.59 ± 0.23	0.54 ± 0.28	0.77 ± 0.17	0.71 ± 0.22	0.72 ± 0.20	0.73 ± 0.18
Syn3	0.77 ± 0.17	0.81 ± 0.14	0.72 ± 0.22	0.72 ± 0.20	0.70 ± 0.24	0.75 ± 0.18

**Table 3:** *t*-test results from three distances (*n* = 528, *α*=0.05).

Synergies	Near	Medial	Far
Syn1	0.79 ± 0.17	0.60 ± 0.26	0.59 ± 0.27
Syn2	0.72 ± 0.19	0.85 ± 0.12	0.77 ± 0.16
Syn3	0.68 ± 0.20	0.73 ± 0.19	0.74 ± 0.18

**Table 4:** Similarity of the synergy among complex motions (CM1 and CM2, CM4 and CM5).

CM2	CM1			CM5	CM4	
	Syn1	Syn2	Syn3		Syn1	Syn2
Syn1	**0.56**	0.25	−0.64	Syn1	−**0.53**	−0.02
Syn2	−0.35	**0.51**	0.30	Syn2	−0.34	**0.73**

**Table 5:** Average of the synergy similarity between complex motions and simple motion.

	CM1	CM2	CM3	CM4	CM5
SM-Syn1	0.74	0.79	0.34	0.69	0.43
SM-Syn2	0.71	0.60	0.74	0.77	0.57
SM-Syn3	0.69	0.85	0.53	0.55	0.81
SM-Syn4	0.71	0.71	0.60	0.87	0.74

**Table 6:** Merging coefficients and reconstructed similarity.

SM-Syn	CM1			CM2		CM3			CM4		CM5	
	Syn1	Syn2	Syn3	Syn1	Syn2	Syn1	Syn2	Syn3	Syn1	Syn2	Syn1	Syn2
SM-Syn1	**0.93**	0.06	0.00	**0.59**	0.02	0.00	0.00	0.00	**0.52**	0.00	0.00	0.00
SM-Syn2	0.00	0.10	**0.81**	0.00	0.25	**0.90**	**0.71**	0.00	0.18	**0.48**	0.00	0.25
SM-Syn3	0.04	**0.58**	0.10	**0.60**	**0.44**	0.00	0.07	**0.64**	0.00	0.07	**0.78**	**0.35**
SM-Syn4	0.11	0.24	0.12	0.00	**0.42**	0.07	0.07	**0.33**	**0.48**	**0.57**	0.26	**0.50**
ReSim	0.98	0.92	0.97	0.98	0.95	0.95	0.84	0.81	0.96	0.97	0.88	0.89

## Data Availability

The raw data used to support the findings of this study are available from the corresponding author upon request.
